# Managing Placenta Accreta Spectrum: A case report on combining conservative care with uterine angioembolization

**DOI:** 10.1016/j.ijscr.2024.109774

**Published:** 2024-05-17

**Authors:** Somayyeh Noei Teymoordash, Sara Ghahari, Sana Movahedi, Zahra Safkhani, Milad Gholizadeh, Soheil Khalili

**Affiliations:** a Department of Obstetrics and Gynecology, School of Medicine, Iran University of Medical Sciences, Tehran, Iran; b Faculty of Medicine, Iran University of Medical Sciences, Tehran, Iran; c Faculty of Medicine, Iran University of Medical Sciences (IUMS), Tehran, Iran; d Department of Pathology, School of Medicine, Iran University of Medical Sciences, Tehran, Iran

**Keywords:** Placenta accreta spectrum, Conservative management, Uterine arterial embolization, Hysterectomy, Case report

## Abstract

**Introduction:**

Placenta Accreta Spectrum (PAS) stands out as one of the most significant complications in pregnancy, capable of causing maternal morbidity and mortality.

**Presentation of case:**

In this report, we aim to discuss a case involving unsatisfactory conservative care coupled with uterine angioembolization, resulting in multiple hospitalizations due to placental infection and eventual hysterectomy.

**Discussion:**

Both conservative and non-conservative approaches have been utilized to mitigate maternal complications and mortality associated with Placenta Accreta Syndrome. While uterus-preserving methods play a crucial role, leaving the placenta in situ can lead to numerous severe long-term complications. Previous Research highlights the limitations of conservative management in the case of placenta accreta, necessitating careful patient selection due to potential morbidity and the risk of secondary hysterectomy.

**Conclusion:**

invasive placentation poses challenges in obstetrics, presenting a risk of severe maternal morbidity and mortality. Conservative management poses limitations and risks, emphasizing the need for further research and evidence-based guidelines to enhance the management of PAS.

## Highlights

• Invasive placentation poses challenges in obstetrics, presenting a risk of severe maternal morbidity and mortality.
• The main goal in treating placenta accreta spectrum (PAS) is to manage hemorrhage, historically addressed with hysterectomy.
• Due to increased risks, there has been a shift toward conservative management.
• Conservative management for placenta accreta has limitations and can lead to complications like organ damage, secondary hysterectomy, and infection.
• Arterial embolization controls surgical morbidity and accelerates placental resorption in the case of retained placenta.

## 1 Introduction

Placenta Accreta Spectrum (PAS) is one of the most concerning complications in pregnancy that can lead to maternal morbidity and mortality. It is a result of the placenta invading the myometrium. PAS encompasses three categories: Accreta, Increta, and Percreta, distinguished by the depth of penetration into the myometrium [1].

Two major PAS risk factors include a history of previous cesarean section and placenta previa, with additional risk factors associated with maternal age over 35, multiparity, uterine scars, and prior intrauterine infections. The incidence of PAS has surged due to the rising rate of cesarean sections [1].

Complications of PAS range from localized uterine wall destruction to severe bleeding, end-organ damage, disseminated intravascular coagulation (DIC), bladder perforation, fistulas, ileus, and lactation failure [2]. Management of PAS involves conservative and non-conservative methods, demanding adequate medical resources, selective management, and comprehensive counseling in well-equipped facilities [3]. In recent decades, the introduction of a conservative approach such as balloon occlusion, methotrexate therapy, and uterine-preserving techniques, which leave placenta in situ emerged as a response to the outcomes associated with hysterectomy [4,5].

While uterus-preserving methods play a crucial role, especially for individuals desiring future pregnancies, leaving the placenta in situ can lead to numerous severe long-term complications. This report discusses a case of unsatisfactory conservative care resulting in multiple hospitalizations for placental infection and eventual hysterectomy. The work has been reported under the SCARE criteria [6].

## 2 Case presentation

An otherwise healthy 36-year-old woman was diagnosed with placenta Percreta and previa at 32 weeks of pregnancy. She was referred to the obstetrics and gynecology department of a tertiary care Hospital in Tehran, Iran, to be managed by a multidisciplinary team.

Upon admission, a comprehensive history was obtained, and a thorough physical examination was conducted. She had no significant past medical history, with a gestational age of 32 weeks, G5P4L2 (gravida 5 para 4 live 2 death 2), including two previous cesarean sections and one preterm delivery due to membrane rupture. She reported no abdominopelvic pain or vaginal bleeding and remained hemodynamically stable. Bimanual and vaginal examinations were not performed, but all other physical examinations were within the normal range.

**Fig. 1:**
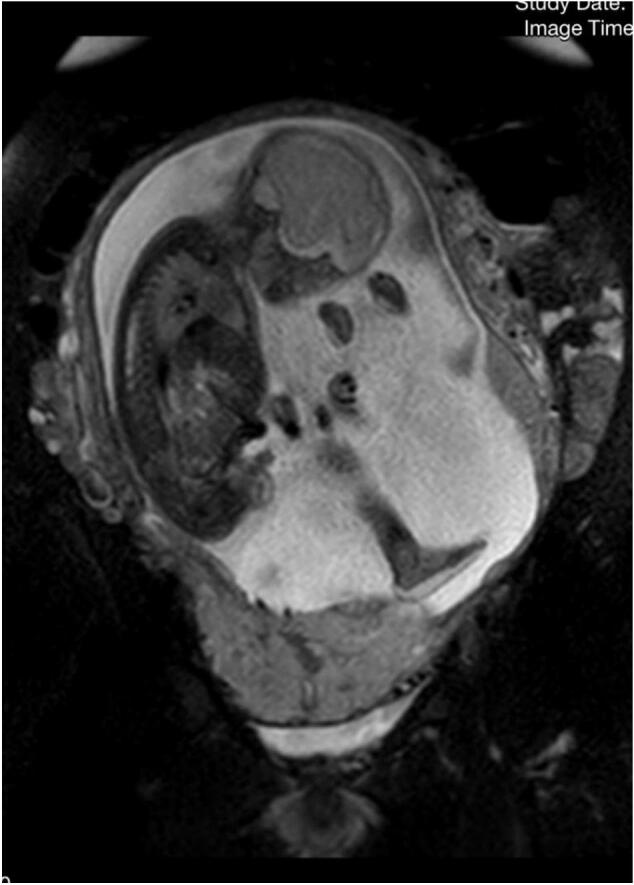
Coronal T2 weighted abdominal magnetic resonance image of invading placenta to the myometrium.

Color Doppler sonography revealed a complete placenta previa with multiple large lacunae, a non-existent hypoechoic retroplacental zone, and abnormal vessels extending from the placenta to the bladder. To assess myometrial invasion of other organs thoroughly, an MRI was performed, revealing abnormal vascularization and lacunae of the placental bed, along with a few areas of intrusion into the posterior wall of the bladder with serosal loss. [Fig. 1] Since the invasion of lacunae to other organs was seen, the case was classified as placenta percreta.

**Fig. 2:**
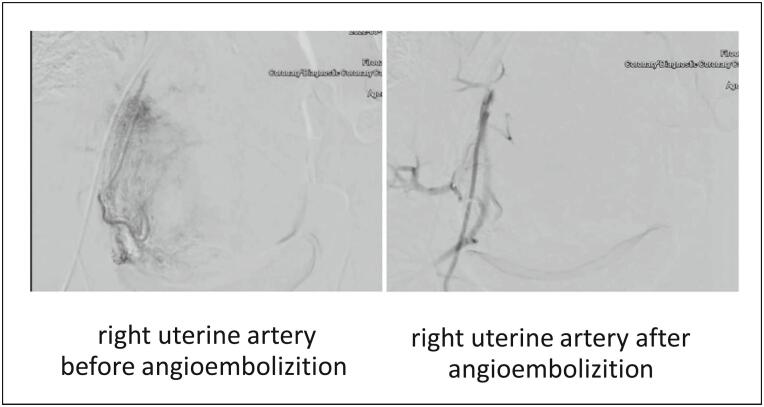
Right uterine artery angiography.

**Fig. 3:**
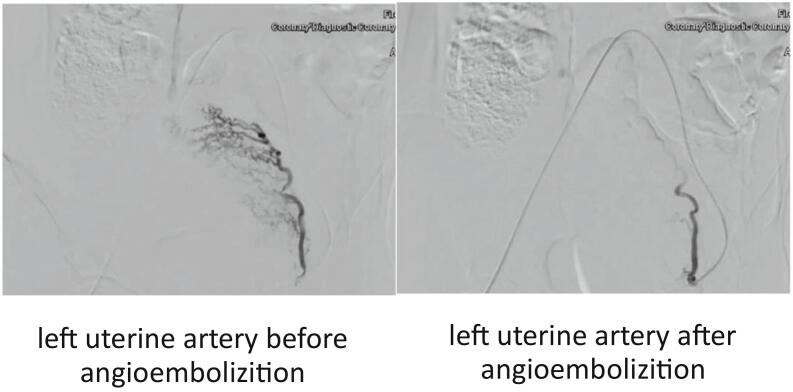
left uterine artery angiography.

Considering the patient's condition, a decision was made to preserve the uterus with the placenta left in situ after delivering the fetus. Following general anesthesia, a midline abdominal incision was made, and a healthy neonate was delivered via a cesarean section. To minimize the risk of hemorrhage and possible nearby organ damage, the umbilical cord and bleeding vessels of the uterus were ligated, and the placenta was left in the uterus. Subsequently, the patient was transferred to the interventional radiology operating room for angioembolization, using polyvinyl alcohol (PVA) particles (500–700 μm) loaded into 10 mL syringes containing a contrast medium. Under Angiographic guidance, a 5-Cobra catheter was placed into both the bilateral uterine artery (UA) and bilateral internal iliac artery (Waltman loop technique for ipsilateral artery). Angiography revealed dilated vascular channels and contrast blush at adhesion sites, which were successfully embolized with PVA until a marked slowing of blood flow was observed. This procedure took about 30–60 min [Fig. 2, Fig. 3].

Post-operatively, antibiotics were administered intravenously for seven days, and the patient was discharged with instructions for weekly complete blood count (CBC) and beta-human chorionic gonadotropin (B-hCG) tests, as well as biweekly color Doppler ultrasonography.

During six months of follow-up, the patient did not resume her menstrual cycle. B-hCG levels gradually decreased and were undetectable after one month. No leukocytosis was observed, and although there was some shrinkage in placental tissue observed during continuous ultrasonography, it was not fully absorbed.

The patient was readmitted to the hospital twice due to abnormal vaginal discharge and tissue disposal. On the last admission, she presented with fever, persistent vaginal bleeding, and malodor vaginal discharge. Ultrasound revealed shrinkage of the placenta and residual heterogenous hypoechoic areas indicative of placental remnants, the bladder wall showed impairment. Laboratory results revealed an elevated white blood cell (WBC) count, erythrocyte sedimentation rate (ESR), and C-reactive protein (CRP). Vaginal culture revealed Acinetobacter colonization.

**Fig. 4:**
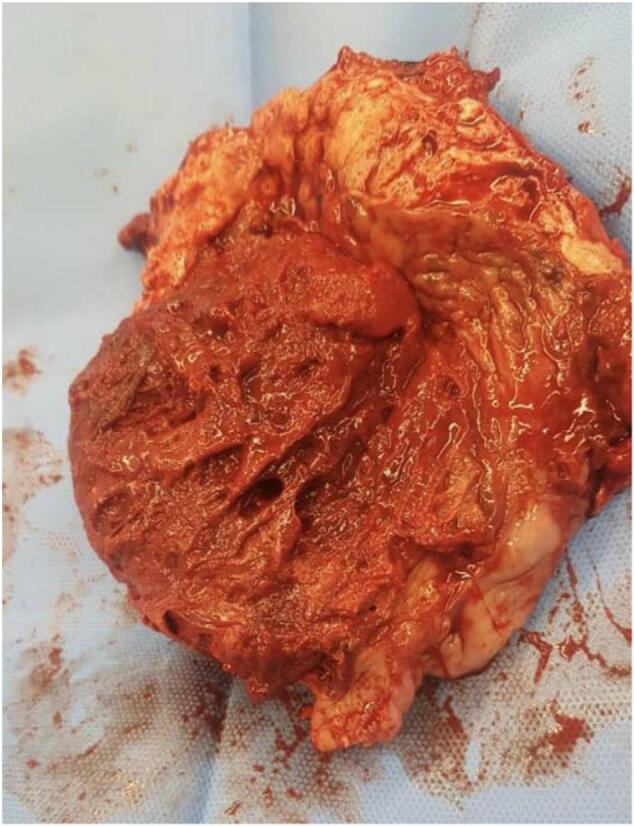
Gross picture of placenta invading myometrium after hysterectomy.

Subsequently, the patient underwent Total Abdominal Hysterectomy (TAH) and Bilateral Salpingectomy, with the urology team assisting in removing and repairing a portion of the bladder dome due to multiple adhesions. [Fig. 4] After the operation, the urethral Foley catheter remained for 10 days, And the patient was discharged in stable condition.

Pathology confirmed the diagnosis of placenta percreta with necrotic tissue.

## 3 Discussion

Invasive placentation poses significant challenges in obstetrics due to the potential for severe maternal morbidity and mortality. In this discussion, we will investigate the failure of conservative management and arterial embolization of placenta percreta, which resulted in multiple hospitalizations due to infection, and ultimately necessitated a hysterectomy 6 months after her C-section.

The primary goal when selecting a treatment strategy for this condition is to manage intrapartum and postpartum hemorrhage (PPH) [3]. Historically, hysterectomy was the preferred treatment for PAS. However, due to its heightened risk of massive PPH and the psychological impact of losing fertility, a conservative approach was introduced. Approximately 50 % mortality and about 7 % morbidity rates have been reported. Thus, over the years, conservative management has gradually replaced the hysterectomy method [1,4,5] In our case, given the high-grade invasion of the placenta through the myometrium and bladder wall, performing a hysterectomy posed a significant risk of severe postpartum hemorrhage and its associated complications.

Consequently, our multidisciplinary team decided on conservative management, which involved leaving the placenta in its original position following cesarean delivery. This approach was augmented by uterine arterial embolization, to facilitate spontaneous absorption of the placenta.

Uterine Arterial Embolization (UAE) is a minimally invasive procedure that blocks the blood supply to the placenta, reducing maternal morbidity and preserving fertility in select cases of invasive placentation [7] Administered by skilled radiologists, arterial embolization is widely accepted as a therapeutic approach for managing bleeding, reducing surgical morbidity, and expediting placental resorption in cases of retained placenta [5].

Other conservative management methods, such as balloon occlusion, methotrexate therapy, and uterine-preserving techniques, are available. Nonetheless, some studies have shown a higher success rate and fewer complications with UAE [3,8,9]. However, in another study, a limited success rate of conservative management using UAE was reported [10].

Conservative management often leads to complications such as secondary hysterectomy, infection, and organ injury in about two-thirds of patients who leave the placenta in situ [3,11] The bladder is the most common organ involved and damaged during both primary TAH and conservative management [11]. Fever is the most frequently reported outcome and usually results from endometritis, sepsis, or an inflammatory reaction to tissue necrosis. The use of prophylactic antibiotics may decrease morbidit [3]. Our patient experienced two episodes of infection despite receiving broad-spectrum antibiotics. Ultimately, due to recurrent infections, she underwent a secondary hysterectomy.

Conservative management necessitates closely monitored follow-up, including the resumption of the menstrual cycle, weekly human chorionic gonadotropin (hCG) levels, and sequential ultrasounds. While our patient's hCG levels declined in the weeks following delivery, placental tissue persisted for a longer duration. Consistent with our findings, hCG levels, which reflect the hormonal activity of the placental tissue, decrease more rapidly than the reduction in visualized tissue remnants [1,12].

Various studies have compared conservative and cesarian treatment. Consistent with our study, a higher risk of maternal morbidity, including infectious diseases, and the need for subsequent surgical intervention was observed in the patients who underwent conservative management [3,11]. However, inconsistent with our findings, some literature reported complete success of conservative management alone [12]. This emphasizes the limitations and potential failures of conservative management in placenta percreta cases.

Conservative management of placenta percreta is a high-risk alternative to surgical treatment, requiring careful patient selection due to potential morbidity and risks of secondary hysterectomy. Lacking proper compliance of patients for follow-up is among the limitations of conservative therapy.

As previously mentioned, having a prior cesarean section is a major risk factor for placenta previa. Our patient had undergone two previous C-sections. Hence, it appears that an effective way to prevent this condition is to avoid unnecessary C-sections Additionally, our patient also had risk factors such as maternal age over 35, multi-parity, and placenta previa. Which could be preventable through public and maternal health education [1,3,8].

To significantly improve the management of placenta percreta, it is imperative to prioritize extensive research endeavors and the formulation of evidence-based guidelines. Such initiatives are paramount in advancing our understanding and optimizing the strategies employed in addressing this complex obstetric condition.

The strength of this research was that all of the patient's imaging was done in one center and it was reported by the same radiologist. The patient might have had problems with recalling her medical history therefore there is a possibility of recall bias in this study.

## 4 Conclusion

While conservative management may initially appear attractive for placenta percreta cases, considering maternal morbidity and mortality, and the absence of standardized guidelines, Healthcare providers must meticulously evaluate individual cases to be able to make informed decisions for optimizing outcomes.

## Ethical approval

Ethical approval for this study was provided by the Iran University of Medical Sciences ethical committee on 15 August 2023. IR.IUMS.REC.1402.26452.

## Funding

This research did not receive any specific grant from funding agencies in the public, commercial, or not-for-profit sectors.

## CRediT authorship contribution statement

All authors contributed to the study, and have read and approved the final manuscript. Noei Teymoordash contributed to data curation and resources and contributed as supervisor. Ghahari wrote, revised, and edited the whole manuscript. Movahedi and Gholizadeh helped with reviewing and editing the manuscript. Safkhani and Khalili helped with some additional resources.

## Guarantor

Sara Ghahari.

## Patient perspective and consent

No identifiable information was disclosed in writing this article. Written informed consent was obtained from the patient for publication of this case report and accompanying images. A copy of the written consent is available for review by the Editor-in-Chief of this journal upon request.

## Declaration of competing interest

The authors declare no conflicts of interest and no external funding. All co-authors have read and approved the manuscript, and no financial interests have been reported.
